# Sarcopenia and its association with objectively measured life-space mobility and moderate-to-vigorous physical activity in the oldest-old amid the COVID-19 pandemic when a physical distancing policy is in force

**DOI:** 10.1186/s12877-022-02861-7

**Published:** 2022-03-25

**Authors:** Rick Yiu Cho Kwan, Justina Yat Wa Liu, Yue-Heng Yin, Paul Hong Lee, Siu Ying Ng, Daphne Sze Ki Cheung, Patrick Pui Kin Kor, Simon Ching Lam, Shirley Ka Lai Lo, Lin Yang, Siu Kay Chan, Vico Chung Lim Chiang

**Affiliations:** 1 School of Nursing, Tung Wah College, Sheung Wan, Hong Kong; 2 Centre for Gerontological Nursing, School of Nursing, The Hong Kong Polytechnic University, Hung Hom, Kowloon, Hong Kong; 3 Research Institute for Smart Ageing, The Hong Kong Polytechnic University, Hung Hom, Hong Kong; 4 Department of Health Sciences, University of Leicester, Leicester, UK; 5 Sik Sik Yuen, Kowloon, Hong Kong; 6 School of Nursing, The Hong Kong Polytechnic University, Hung Hom, Kowloon, Hong Kong

**Keywords:** Sarcopenia, Moderate-to-vigorous physical activity, Life-space mobility, COVID-19, Physical distancing

## Abstract

**Introduction:**

The oldest-old are highly vulnerable to sarcopenia. Physical distancing remains a common and effective infection-control policy to minimize the risk of COVID-19 transmission during the pandemic. Sarcopenia is known to be associated with impaired immunity. Moderate-to-vigorous physical activity (MVPA) and life-space mobility (LSM) are potential strategies for minimizing the risk of sarcopenia. However, a physical distancing policy might jeopardize the practice of MVPA and LSM. The purposes of this study were to identify the prevalence of sarcopenia and examine the association between MVPA and LSM with sarcopenia in the community-dwelling oldest-old during the COVID-19 pandemic.

**Methods:**

This study employed a cross-sectional and observational design. The study was conducted in 10 community centres for older people in Hong Kong during the period of the COVID-19 pandemic (September to December 2020). Eligible participants were the oldest-old people aged ≥85 years, who were community-dwelling and had no overt symptoms of cognitive impairment or depression. Key variables included sarcopenia as measured by SARC-F, LSM as measured by a GPS built into smartphones, and MVPA as measured by a wrist-worn ActiGraph GT3X+. Variables were described by mean and frequency. A multiple linear regression was employed to test the hypotheses. The dependent variable was sarcopenia and the independent variables included LSM and MVPA.

**Results:**

This study recruited 151 eligible participants. Their mean age was 89.8 years and the majority of them were female (*n* = 93/151, 61.6%). The prevalence of sarcopenia was 24.5% (*n* = 37/151) with a margin of error of 6.86%. MVPA was negatively associated with sarcopenia in older people (*β* = − 0.002, SE = 0.001, *p* = 0.029). However, LSM was not associated with sarcopenia.

**Conclusion:**

The prevalence of sarcopenia in the community-dwelling oldest-old population is high. MVPA is negatively associated with sarcopenia. LSM is unrelated to sarcopenia. Sarcopenia should be recognized and the oldest-old with sarcopenia should be accorded priority treatment during the COVID-19 pandemic.

**Supplementary Information:**

The online version contains supplementary material available at 10.1186/s12877-022-02861-7.

## Introduction

Sarcopenia is defined by the European Working Group on Sarcopenia in Older People (EWGSOP) as a reduction in appendicular skeletal muscle mass, muscle strength, and physical performance [1]. The prevalence of sarcopenia in older people ranges from 0.36 to 8.14%, varying according to the diagnostic criteria [2]. Sarcopenia is a disease linked to other diseases; thus, its prevalence is much higher in older people with chronic illnesses; for example, 42% of people with stroke, 31.4% of people with cardiovascular diseases, and 26.4% of people with dementia have sarcopenia [3, 4]. Recent evidence shows that older age is associated with a higher risk of developing sarcopenia and that the oldest-old with sarcopenia have higher odds of experiencing difficulties with physical function (e.g., stooping, kneeling, crouching, walking) [5]. Sarcopenia is also associated with a higher risk of many negative health outcomes, including death from all causes, in-hospital mortality, and falls [6].

Many countries have adopted physical distancing and stay-at-home policies as key strategies to slow the spread of the virus since the World Health Organization (WHO) declared coronavirus disease 2019 (COVID-19) to be a global pandemic in March 2020 [7, 8]. As in other regions during this pandemic, the Hong Kong Government adopted a physical distancing policy, advising the public to reduce their level of social contact. Specifically, people were instructed to maintain a distance of at least 1 m from others; minimize gatherings, group-based activities (e.g., fitness classes), and trips outside the home (particularly meal gatherings); avoid crowded places and physical contact (e.g., handshakes); and wear face masks [9]. Hong Kong people have shown good compliance with these infection-control measures [10, 11]. However, the prolonged implementation of the physical distancing and stay-at-home policies could increase the risk of people of all ages developing a variety of health problems [12]. Research has shown that older people with chronic conditions greatly decreased their physical activity levels and were more likely to be hospitalized during the pandemic [13, 14]. Older people, particularly the oldest-old (i.e., those aged over 85 years), may be more susceptible to the negative consequences of rigorous distancing [15].

It has been recommended that older people engage in at least 150 min of moderate-to-vigorous physical activity (MVPA), at 10-min bouts each time for beneficial health outcomes [16]. MVPA is associated with many health benefits (e.g., reduced mortality) [17]. A significant reduction in physical activity levels worldwide has been identified as a negative impact of physical distancing [18]. A study showed that 2 weeks of inactivity could result in decreased muscle mass strength in approximately 8% of people, leading to ineffective muscle function [19]. An abrupt reduction in physical activity because of physical distancing is a particular concern for older people, [20] as all of these physiological changes may predispose older people to frailty and sarcopenia [21].

Life-space mobility (LSM) refers to movement extending from within one’s household to beyond one’s district or town [22]. Previous studies have demonstrated an association between declining life-space and an increased risk of frailty, [23] reduced physical activity, [24] and poor mental health [25]. A few studies have reported a significant reduction in LSM among older people during the COVID-19 pandemic [26, 27]. Studies conducted in Brazil and Finland demonstrated that restrictions in life-space during the COVID-19 pandemic had a negative impact on the quality of life of older people [15, 28].

Physical activity and engagement in activities outside the home are recommended treatments for sarcopenia in older people [29]. However, the LSM measured in studies of sarcopenia in older people has commonly been evaluated using questionnaires such as Life-Space Assessment [22]. Accuracy affected by the recall bias of participants is the major limitation of using a questionnaire to measure LSM [30]. Furthermore, there is a dearth of studies examining the associations among sarcopenia, physical activity, and LSM during the COVID-19 pandemic when strict infection-control policies (e.g., physical distancing, a stay-at-home policy) were in force; not to mention studies focusing on the most vulnerable older people (i.e., the oldest-old). This study will inform policymakers on how to sustain health in the oldest-old during the COVID-19 pandemic.

## Objectives

This study aims to:

1. Estimate the prevalence of sarcopenia in the oldest-old during the COVID-19 pandemic, and
2. Examine the association between sarcopenia with objectively measured MVPA and LSM.

## Methods

### Study design

This study employed a cross-sectional and observational design. To ensure clarity of reporting, we followed the Strengthening the Reporting of Observational Studies in Epidemiology (STROBE) guidelines [31].

### Setting

The study was conducted in ten community centres for older people in Hong Kong. The centres provide a range of activities for community-dwelling people aged ≥60 years to enable them to remain in the community and to lead healthy and dignified lives [32]. We adopted a convenience sampling method to invite community centres to join the study. The staff members in the community centres were briefed on the sample selection criteria. They then invited those of their members who met the criteria to be screened by our research team. The study, including recruitment and data collection, was conducted from September to December 2020. During this period, Hong Kong was experiencing the COVID-19 pandemic, with a mean daily number of 26 new cases (range = 0–115, median = 8) in a city with a population of approximately 7.5 million [33].

### Participants

Inclusion criteria were 1) the oldest-old people aged ≥85 years, [34] and 2) community-dwelling, defined as not having lived in a long-term care setting (e.g., nursing home) in the past 6 months before the data collection process. Participants were excluded if they were in a condition that could hinder them from providing accurate information, including 1) cognitive impairment, defined by a Mini-Cognitive Test score of ≥3, [35] and depressive symptoms, defined by a Geriatric Depression Scale score of ≥8 [36, 37].

We asked staff members of the community centres to invite all potentially eligible participants to join the study through face-to-face and telephone invitations. Potentially eligible participants were then invited to attend the eligibility screening sessions, which were conducted in the community centres.

### Variables

Demographic variables included age, gender, marital status, educational attainment, and living conditions. Self-reporting was used to collect the demographic information of the participants, such as their living conditions, including type of residence and with whom they were living; and level of education, including the highest level of education that they had completed. Clinical variables included chronic illnesses, body mass index (BMI), and cognitive function. Predictor variables included life space and MVPA. Outcome variables included sarcopenia. Data collection was conducted at the community centres by a trained research assistant through face-to-face interviews using validated instruments.

### Measurement

Chronic illnesses were measured using a questionnaire with six dichotomous questions (0 = no, 1 = yes) on common chronic illnesses, including Diabetes Mellitus, cardiovascular diseases, osteoarthritis, cancer, and hypertension, which were found to be associated with sacropenia [4, 38].

Body mass index (BMI) was measured using a calibrated scale balance and a tape ruler employing the standard eq. (BMI = body weight in kg^2/body height in metres). BMI was categorized into four levels adjusted for Asian adults, as recommended by the WHO Western Pacific Region (underweight: BMI < 18.5, normal: BMI = 18.5–22.9, overweight: BMI = 23–25, and obese: BMI > 25) [39].

Cognitive function was measured using the Abbreviated Mental Test (AMT) Hong Kong version [40]. AMT consists of 10 items evaluating orientation to time and place, attention, calculation, and memory. One point is assigned for a correct answer to each question, with a total score ranging from 0 to 10. A higher score indicates better cognitive function. It has been validated to have good internal consistency (α = 0.814), test-retest reliability (r = 0.993), and concurrent validity with the Chinese Mini-Mental Stage Examination (r = 0.86) [40, 41].

Sarcopenia was measured using the SARC-F questionnaire [42, 43]. SARC-F comprises five items assessing strength, assistance in walking, rising from a chair, climbing stairs, and falls. Each item was rated on a 3-point frequency scale (i.e., 0 = none to 2 = A lot) with a total score ranging from 0 to 10. A higher score indicates a higher risk of sarcopenia. It has been validated against the gold standard criteria of EWGSOP to have good validity in identifying sarcopenia (accuracy = 0.90–0.87) [43].

**Fig. 1:**
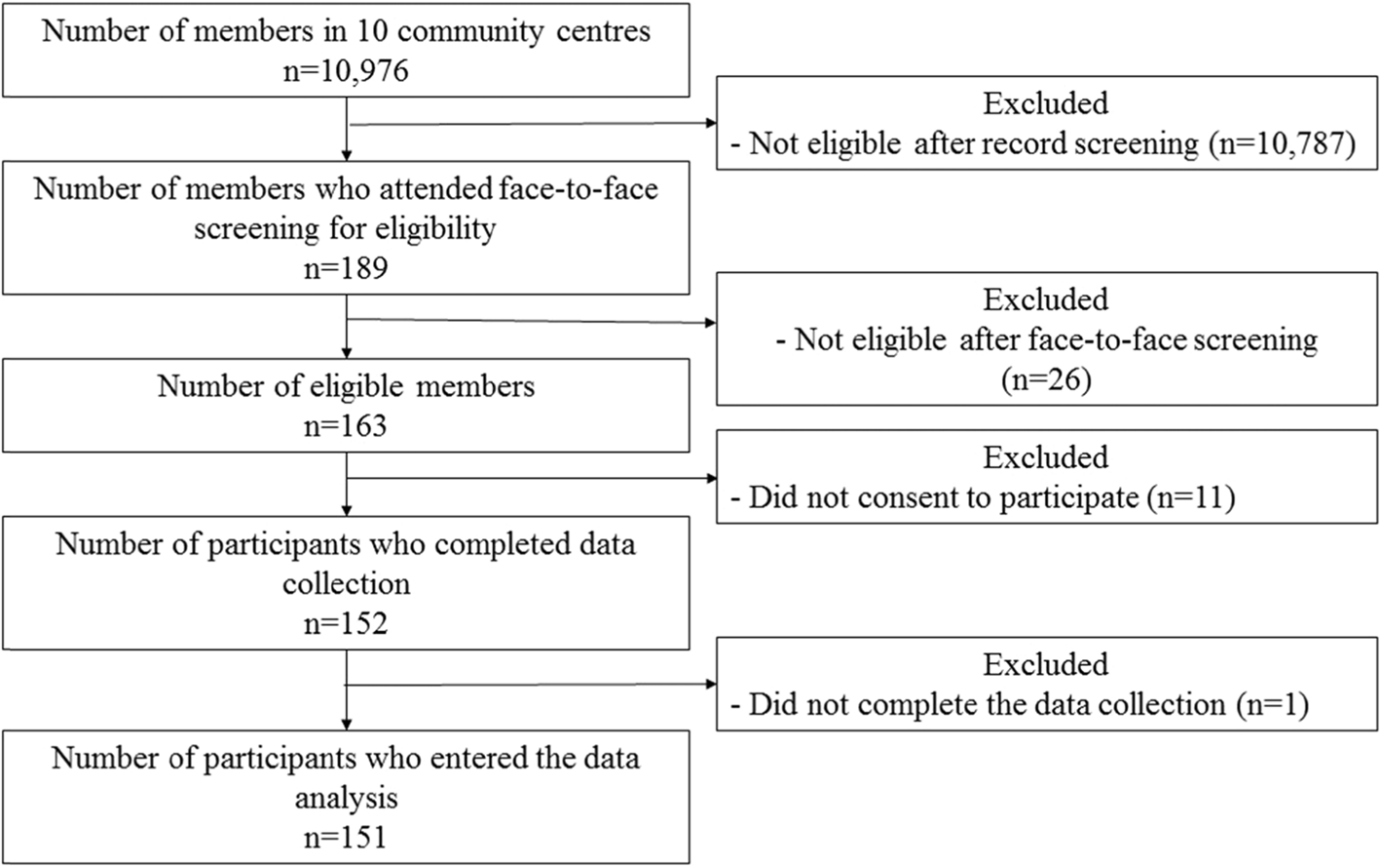
Flowchart of the Data Collection Process

In this study, LSM is conceptualized as the area through which a person has travelled over a specific period, expanding from within one’s home to beyond one’s town or geographic region [44]. Therefore, LSM was measured using an objective measurement employing an Android-based smartphone (i.e., Redmi 9, Xiaomi) installed with a global positioning system (GPS) logging app. The location of the participants was collected using the Application Programming Interface (APK) provided by Google Maps. Data collection was automatically triggered every 15 min using Android Job Scheduler. As shown in Fig. 1, the participants were instructed to carry the smartphones with them continuously using the pouches provided. GPS is becoming more ubiquitous every day, as most smartphones have a built-in GPS function. GPS uses a person’s geolocation to calculate various measures related to LSM, such as frequency and distance travelled from home. Some studies using GPS to determine LSM have reported promising results [45, 46]. The acceptability and feasibility of having older people use GPS has also been found to be satisfactory [47, 48]. Participants were asked to carry the smartphone with GPS at all times for 7 days.

GPS coordinates were categorized into five zones following the conceptualization of the Life Space Assessment (LSA) instrument validated in a Chinese older population [49]. Categorization of the coordinates into the five zones depended on the distance of the coordinates from the residential addresses of individual participants [50]. Given that GPS could not accurately differentiate between a bedroom and an apartment, we collapsed the zones of the bedroom and within the apartment building. We adopted a 4-concentric-circle categorization, with the centres at the participants’ residential address (i.e., zone 1 = bedroom/within the apartment building, zone 2 = neighbourhood other than own yard or apartment building, zone 3 = outside the neighbourhood but within the town, and zone 4 = outside the town). Based on a previous study, zone 1 was defined as the area of the concentric circular zone with a radius of 0–150 m, while zone 4 was defined as the area beyond the circular zone with a radius of 1000 m [50]. The cut-off points for zones 2 and 3 were estimated as being half of the difference between the radii of zones 1 and 4 (i.e., [150–1000 m]/2). Thus, zones 2 and 3 were the circular areas with radii of 51–580 m and 581–1000 m, respectively. The percentage of time spent in each zone over the total amount of time spent in collecting data was calculated. An LSM score was computed by summing the weighted percentage of time spent in each zone. The weighting was assigned according to the distance of the zone from the participant’s home (i.e., zone 1 = 1.5, zone 2 = 3, zone 3 = 4, and zone 4 = 5). The weighting assigned to each zone refers to the weighting used in the Life Space Questionnaire [49]. A higher LSM score indicates a higher level of LSM. Details regarding the smartphone wearing compliance and GPS logging methods could be found in the Supplementary Materials.

MVPA was measured using a wrist-worn ActiGraph GT3X+, which was mounted on the participants’ non-dominant wrist for 24 h for 7 days. ActiGraph GT3X+ was validated against indirect calorimetry to accurately identify MVPA (sens = 0.836, spec = 0.894, AUC = 0.932). Participants were instructed to wear the device continuously during the assessment period [51]. An MVPA minute was defined as a minute in which the ActiGraph recorded physical movement (i.e., vector magnitude) of above 4117.7 cpm [51]. Only at least 10 min of continuous MVPA were counted as valid minutes because only such sessions are considered beneficial by the WHO [16]. MVPA minutes were measured over seven consecutive days with an epoch length of 1 minute [52]. Participants were instructed to wear the ActiGraph continuously throughout the 7-day study period (i.e., 24 h/day for 7 days). We used 60 min of a continuous count of zero to determine “non-wear time” [53]. Only MVPA minutes measured on valid days (i.e., ActiGraph wear time > 10 h/day) for a valid period (i.e., valid days ≥4 days) were entered into the data analysis [54, 55].

### Study size

To estimate the sample size needed to determine the prevalence of sarcopenia in the oldest-old (i.e., objective #1), Cochran’s formula was employed [56]. We assumed that the prevalence of sarcopenia was 7.3%, as observed in a local study of community-dwelling older people in Hong Kong [57]. Cochran’s formula showed that 163 participants were needed, with a confidence interval of 95% and a margin of error of 4% in the population of the oldest-old (i.e., people aged ≥85 years) of approximately 170,000 people, as estimated in the 2016 Hong Kong census [57].

To estimate the sample size needed to determine the association between sarcopenia and MVPA minutes and LSM, the following linear multiple regression was employed: Fixed model, R^2^ increase package in G*Power. The effect size of accelerometer-determined MVPA minutes on sarcopenia was small-medium (i.e., Cohen’s d = 0.47) [58, 59]. We did not consider the effect size of LSM on sarcopenia because this information was not available in the literature. G*Power showed that 158 participants would be needed, with the parameters estimated to be f^2^ = 0.1, α = 0.05, power = 0.95, number of tested predictors = 2, and the total number of predictors = 14.

To ensure an adequate number of samples to fulfil the two research objectives, we adopted a sample size of 163.

### Statistical methods

Demographic variables, clinical variables, and outcomes were described using mean and frequency according to their levels of measurement. To test the association between sarcopenia with LSM and physical activity (i.e., objective #2), multiple linear regression was employed. The dependent variable was sarcopenia. The independent variables were LSM and MVPA. Potential confounding variables included age, gender, education, chronic diseases, cognitive function, living condition, and BMI because they are known to be associated with sarcopenia, as well as LSM and physical activity [6, 28].

Missing data on the demographic and clinical variables were replaced by mean values. Missing MVPA data were defined as those detected as non-wearing, and missing GPS data were defined as data collected within an invalid period or collected with poor accuracy. Both GPS and MVPA data were replaced by mean values, and only available data on valid days were entered into the data analysis [54, 55]. A sensitivity analysis comparing results between two missing data management strategies (i.e., replacement by mean) was performed.

## Results

### Participants

As shown in Fig. 1, we recruited participants in 10 community centres for older people. We assessed the eligibility of 189 members of these centres. In the pre-screening phase, 10,976 members were identified as not eligible to take part in the study, through a reading of their records in the centres by centre staff. Common reasons for exclusion included age, a documented condition of dementia, and an inability to speak Cantonese. We identified 163 eligible participants, but 11 did not consent to data collection and 1 did not complete the data collection process. In the end, we included data from 151 participants for analysis.

There were no missing data on the demographic and clinical variables, except on BMI, which was replaced by the mean value. There were 32 invalid cases (21.2%) of Actigraph data and 26 invalid cases (17.2%) of GPS data; these were replaced by the mean values of other participants. There were no significant differences in missing data between the groups with and without sarcopenia.

### Descriptive data

**Fig. 2:**
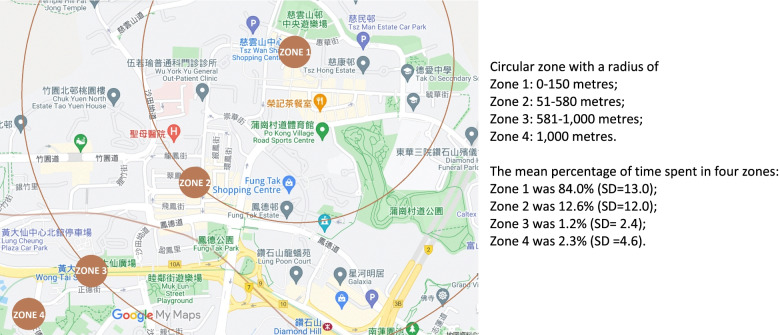
The LSM demonstrated with GPS coordinates

As shown in Table 1, the participants’ mean age was 89.8 years. The majority were female (*n* = 96, 63.6%), widowed/divorced/separated (*n* = 93, 61.6%), uneducated (*n* = 79, 52.3%), living with family/domestic helper/friends (*n* = 88, 57.9%), had hypertension (*n* = 108, 71.5%), and normal BMI (*n* = 61, 40.4%). The mean AMT score was 8.5 (SD = 1.3). The mean SARC-F score was 2.1 (SD = 2.1). The mean number of MVPA minutes per week was 181.3 (SD = 202.5). The mean LSM score was 1035.3 (SD = 141.7). The mean percentage of time spent in zone 1 was 84.0% (SD = 13.0), in zone 2 was 12.6% (SD = 12.0), in zone 3 was 1.2% (SD = 2.4), and in zone 4 was 2.3% (SD = 4.6) (Fig. 2).

**Table 1:** Participants’ demographic and clinical profile, predictors, and outcomes (*N* = 151)

*Demographic and clinical profile*	mean (SD) / n (%)
Age, mean year (SD)	89.8 (3.6)
Gender, n (%)	
Male	55 (36.2)
Female	96 (63.6)
Marital status, n (%)	
Unmarried	2 (1.3)
Married	56 (36.8)
Widowed/divorced/separated	93 (61.6)
Educational attainment, n (%)	
Tertiary	2 (1.3)
Secondary	13 (8.6)
Primary	63 (41.7)
Nil	73 (48.3)
Living condition, n (%)	
Living alone	63 (41.7)
Living with family/domestic helper/friends	88 (57.9)
Chronic diseases, n (%)	
Diabetes Mellitus	21 (13.9)
Heart diseases	26 (17.2)
Osteoarthritis	8 (5.3)
Cancer	5 (3.3)
Hypertension	108 (71.5)
BMI, mean (SD)	23.6 (3.7)
BMI, n (%)	
Underweight (BMI < 18.5)	8 (5.3)
Normal (BMI = 18.5–22.9)	61 (40.4)
Overweight (BMI = 23–25)	36 (23.8)
Obese (BMI > 25)	46 (30.5)
Cognitive function	
AMT, mean (SD)	8.5 (1.3)
*Predictors*	
MVPA	
Minutes per week, mean (SD)	181.3 (202.5)
Life-space mobility score, mean (SD)	1035.3 (141.7)
Time spent in each life-space zone, mean % (SD)	
Zone 1	84.0 (13.0)
Zone 2	12.6 (12.0)
Zone 3	1.2 (2.4)
Zone 4	2.3 (4.6)
*Outcomes*	
Sarcopenia (SARC-F), mean (SD)	2.1 (2.1)
Sarcopenia (SARC-F ≥ 4), n (%)	
Yes	37 (24.5)
No	114 (75.5)

*BMI* body mass index, *AMT* Abbreviated Mental Test, *MVPA* moderate-to-vigorous physical activity, *Zone 1* bedroom/within the apartment building, *Zone 2* neighbourhood other than own yard or apartment building, *Zone 3* outside neighbourhood but within the town, *Zone 4* outside the town, *SARC-F* SARC-F questionnaire

### Main results

#### Objective #1

The prevalence of sarcopenia was 24.5% (*n* = 37/151) with a margin of error of 6.86%.

#### Objective #2

As shown in Table 2, MVPA was negatively associated with sarcopenia in older people (*β* = − 0.002, SE = 0.001) and the association was statistically significant (*p* = 0.047). However, the LSM score was not associated with sarcopenia. Age and cognitive function were also observed to be associated with sarcopenia. These factors explained 27% of the variance for sarcopenia (*R*^*2*^ = 0.270). A sensitivity analysis did not reveal any differences in the interpretation of the results between the two missing data management strategies (i.e., list-wise deletion and replacement by mean).

**Table 2:** Regression model predicting sarcopenia by SARC-F score

*N* = 151, *R*^2^ = 0.295	Beta (SE)	*p*-value	95%CI
*Confounding factors*			
Gender			
Female	1.573 (0.403)	< 0.001*	0.776, 2.371
Male	0		
Age	0.017 (0.049)	0.733	−0.080, 0.114
Education			
Tertiary	−1.820 (1.395)	0.149	−4.579, 0.940
Secondary	−0.232 (0.618)	0.708	−1.454, 0.990
Primary	−0.290 (0.359)	0.421	−1.002, 0.421
Nil	0		
Cognitive function (AMT)	−0.333 (0.133)	0.013*	−0.595, − 0.070
Marital		0.141	−0.055, 0.380
Unmarried	0.432 (1.395)	0.757	−2.328, 3.192
Married	0.074 (0.409)	0.856	−0.735, 0.883
Widowed/divorced/separated	0		
Living condition			
Living alone	−0.391 (0.352)	0.270	−1.088, 0.307
Living with family/domestic helper/friends	0		
BMI	0.062 (0.045)	0.177	−0.028, 0.151
Chronic diseases			
Diabetes Mellitus	−0.019 (0.476)	0.968	−0.961, 0.923
Heart diseases	0.045 (0.425)	0.915	−0.795, 0.886
Osteoarthritis	0.783 (0.702)	0.267	−0.606, 2.172
Cancer	0.117, (0.894)	0.896	−1.652, 1.887
Hypertension	−0.118 (0.375)	0.753	−0.860, 0.623
*Predictors*			
MVPA minutes per week	−0.002 (0.001)	0.047*	−0.003, −2.572
Life Space Mobility	0.038 (0.052)	0.464	−0.064, 0.140
Time spent in each life-space zone			
Zone 1	44.499 (67.279)	0.509	−88.568, 177.566
Zone 2	6.456 (44.623)	0.877	−75.866, 88.778
Zone 3	−21.628 (44.954)	0.631	−110.539, 67.282
Zone 4	−40.724 (62.422)	0.515	− 164.184, 82.735

## Discussion

This appears to be the first study to examine the association between sarcopenia with objectively measured MVPA and LSM in community-dwelling oldest-old people. There are three key findings. First, the prevalence of sarcopenia in the oldest-old during the COVID-19 pandemic was high. Second, MVPA was negatively associated with sarcopenia in the oldest-old. Third, the MVPA of the majority of the oldest-old was adequate (i.e., MVPA min > 150 min/week) even though the participants did not go far from their residence. These findings have several implications.

A study that surveyed 670 older people in the Chinese community with a mean age of 76.2 years, and employing the same method (i.e., SARC-F score ≥ 4), showed that the prevalence of sarcopenia was 6.1% [60]. A systematic review of seven studies (*n* = 12,800) that included older people with a mean age of 75.1 years and that employed various diagnostic criteria (e.g., EWGSOP, International Working group on Sarcopenia, Asian Working Group for Sarcopenia, and Foundation for the National Institutes of Health Sarcopenia Project) showed that the prevalence of sarcopenia in older people was 3–17.5% [61]. A study examining the prevalence of sarcopenia specifically in the oldest-old community-dwelling Chinese showed that the prevalence increased dramatically from 15.1% in those aged 80–84 years to 63.6% in those aged 95+ years [5]. In comparison, the percentage of older people with sarcopenia observed in this study was lower (i.e., 24.5%). A possible reason for why more older people in our study were observed to have sarcopenia is that the effects of COVID-19 (e.g., confinement on physical activity, dietary habits, sleep, and stress) might have given rise to an increased likelihood of muscle loss [21]. The high prevalence of sarcopenia could also be due to the age of the sample in this study, which included only the oldest-old. The prevalence of sarcopenia is known to increase exponentially with age [5]. This study suggests that COVID-19 prevention measures (e.g., lockdown) might have led to more nutrition problems, reduced physical activity, and increased social isolation, all of which would lead to sarcopenia. The oldest-old are a particularly vulnerable segment of the population. Sarcopenia could subsequently induce impaired immune function and heightened metabolic stress, [62] leading to susceptibility to COVID-19 infection. Therefore, this study recommends that sarcopenia be recognized and treated as a priority during the COVID-19 pandemic.

This study adopted the approach of including all forms of MVPA that last continuously for 10 min in a free-living setting. Given that it could be difficult for older people in free-living settings to recognize MVPA, it is recommended that they wear trackers to help to ensure that they attain an intensity consistent with the definition of MVPA when they exercise. This is because a systematic review showed that commercially available wearable trackers are valid for measuring MVPA [63]. Therefore, this study recommends that the oldest-old practise MVPA in any form for 10 continuous minutes gauged by wearable trackers to combat sarcopenia during the COVID-19 period. For example, brisk walking in a free-living setting could be intense enough to achieve MVPA and could feasibly be practised by older people in free-living settings [64].

A recent study conducted in a Chinese population with a mean age of 70 years before the COVID-19 pandemic showed that older people spent an average of 175.0 min in MVPA per week [65]. This is very similar to what we observed in this study (i.e., 181.3 min/week). This shows that the amount of MVPA practised by the oldest-old is similar to the average for older people and has been unaffected by the COVID-19 pandemic. The average amount of MVPA in the oldest-old is above the beneficial amount of 150 min/week as recommended by the WHO. The GPS data indicated that the vast majority of participants did not go far from their apartments. MVPA is unlikely to be achieved at home given the small living spaces in Hong Kong. This study showed that it was feasible to remain physically active without going far from home. Also, despite the adequate amount of MVPA on average, the prevalence of sarcopenia was still somewhat high. Future studies should examine what other factors contributed to the higher level of sarcopenia in the oldest-old during the COVID-19 pandemic. This study, therefore, recommends that the oldest-old remain physically active (i.e., MVPA > 150 min/week) during the COVID-19 pandemic, pay attention to other risk factors of sarcopenia apart from inadequate physical activity (e.g., poor nutrition), and avoid unnecessary travel to minimize the risk of contracting COVID-19 [66].

This study has several limitations. First, the sample size was relatively small. Caution should be exercised on the claim of the prevalence of sarcopenia because the margin of error is not low (i.e., 6.86%). Second, although we had already conducted subject screening and recruitment at 10 community centres, those were convenience samples and no sampling frame was used. Caution should also be exercised on the issue of the generalizability of the findings to the population. Third, there is a notable amount of missing data relating to the MVPA and GPS. Yet a sensitivity analysis showed no differences in interpretation of the results between the two management methods (i.e., replaced by mean values and listwise deletion). Fourth, because of the short battery life of the smartphone, GPS data could not be mandatorily sampled every 15 min. Instead, the GPS data copied the previous coordinate every 15 min if no movement was detected. The “no movement” assumption could also be a case of poor reception of GPS signals. Fifth, the effect size of MVPA on sarcopenia was observed to be very strong, and significant. Sixth, the SARC-F has low sensitivity, so we might have dismissed positive cases, meaning that the actual prevalence of sarcopenia might be higher. But the SARC-F is still an effective tool for screening potential cases of sarcopenia because of its high specificity (relatively good overall diagnostic accuracy) [43]. Finally, there is the possibility of an undetected residual confounding bias, so the findings need to be treated dialectically.

## Conclusion

The prevalence of sarcopenia in the community-dwelling oldest-old population is high. MVPA is negatively associated with sarcopenia. LSM is unrelated to sarcopenia. During the COVID-19 pandemic, sarcopenia should be recognized and treated as a priority for the oldest-old.

## Supplementary Information

**Additional file 1.**

## Data Availability

The datasets generated and analysed during the current study are not publicly available due to the restrictions involved when obtaining ethical approval for our study, which commit us to sharing the data only with members of the research team, but allow data to be made available from the corresponding author upon reasonable request.

## Abbreviations

COVID-19: Coronavirus Disease of 2019
MVPA: Moderate-to-Vigorous Physical Activity
LSM: Life-Space Mobility
SARC-F: Strength, Assistance with walking, Rising from a chair, Climbing stairs, and Falls
EWGSOP: European Working Group on Sarcopenia in Older People
WHO: World Health Organization
STROBE: Strengthening the Reporting of Observational Studies in Epidemiology
BMI: Body Mass Index
AMT: Abbreviated Mental Test
GPS: Global Positioning System
APK: Android Application Package
AGPS: Assisted Global Positioning System
GLONASS: Global Navigation Satellite System
