# Supplementary material for: Sarcopenia and its association with objectively measured life-space mobility and moderate-to-vigorous physical activity in the oldest-old amid the COVID-19 pandemic when a physical distancing policy is in force
Source: BMC Geriatr. 2022 Mar 25;22:250. doi: 10.1186/s12877-022-02861-7 (PMC8956332; doi:10.1186/s12877-022-02861-7)
Supplement: Supplementary file 1 — Additional file 1. [file 12877_2022_2861_MOESM1_ESM.docx]

# Supplementary materials

Their compliance was monitored by combining the GPS data and step counts. In addition, a trained research assistant made regular calls to the participants to remind them to carry the phones. Two types of location sources, namely GPS receivers (including GPS, AGPS, GLONASS, and Baidu) and a 4G network, were used for receiving GPS signals. Since all of these location providers were running at the same time when locating the participants, Google combined the results from multiple providers to determine the data for each location.(1) The accuracy of the identified location was indicated by the radius (in metres) of a circle, with the true location of the participants being inside the area of the circle.(2) The GPS data collected using a Xiaomi smartphone was also empirically criterion-validated with the geodetic GPS receiver, which showed that the accuracy was at the decimetre level.(3)

Location coordinates (i.e., latitude and longitude) were continuously logged by the smartphone at a sampling interval of 15 minutes. To save battery power, logging stopped when no movement was detected by the smartphones. However, mandatory logging by the app took place every 24 hours. When the mandatory logging of data was not observed on two consecutive days and no GPS data could be logged by the app between these two time points of mandatory logging, this indicated that the app could not sample GPS data for some reason (e.g., malfunctioning of the app or no GPS signals). Those two days were regarded as invalid. If a case was not regarded as valid, the GPS data of the participant was not entered into the data analysis. Only GPS data measured on valid days (i.e., mandatory logs observed on two consecutive days) for a valid period (i.e., ≥4 days) were entered into the data analysis.

**References**

1. Google. Location Request 2021 [updated 18 February, 2021. Available from: <https://developers.google.com/android/reference/com/google/android/gms/location/LocationRequest.html>.

2. Google. Location 2021 [updated 9 June, 2021. Available from: <https://developer.android.com/reference/android/location/Location>.

3. Elmezayen A, El-Rabbany A. Precise Point Positioning Using World’s First Dual-Frequency GPS/GALILEO Smartphone. Sensors. 2019;19(11) doi:10.3390/s19112593.
